# Ionizing Radiation for Preparation and Functionalization of Membranes and Their Biomedical and Environmental Applications

**DOI:** 10.3390/membranes9120163

**Published:** 2019-12-03

**Authors:** Maria Helena Casimiro, Luis Mota Ferreira, João Paulo Leal, Claudia Cristina Lage Pereira, Bernardo Monteiro

**Affiliations:** 1 Centro de Ciências e Tecnologias Nucleares (C2TN), Instituto Superior Técnico, Universidade de Lisboa, Campus Tecnológico e Nuclear, Estrada Nacional 10, ao km 139,7, 2695-066 Bobadela, Portugal; 2 Centro de Ciências e Tecnologias Nucleares (C2TN), Departamento de Engenharia e Ciências Nucleares (DECN), Instituto Superior Técnico, Universidade de Lisboa, Campus Tecnológico e Nuclear, Estrada Nacional 10, ao km 139,7, 2695-066 Bobadela, Portugal; ferreira@ctn.tecnico.ulisboa.pt (L.M.F.); jpleal@ctn.tecnico.ulisboa.pt (J.P.L.); 3 Centro de Química Estrutural (CQE), Instituto Superior Técnico, Estrada Nacional 10, 2695-066 Bobadela, Portugal; 4 LAQV-REQUIMTE, Departamento de Química, Universidade Nova de Lisboa, 2829-516 Monte de Caparica, Portugal; ccl.pereira@fct.unl.pt

**Keywords:** radiation technologies, membranes, polymers, environmental applications, biomedical applications

## Abstract

The use of ionizing radiation processing technologies has proven to be one of the most versatile ways to prepare a wide range of membranes with specific tailored functionalities, thus enabling them to be used in a variety of industrial, environmental, and biological applications. The general principle of this clean and environmental friendly technique is the use of various types of commercially available high-energy radiation sources, like ^60^Co, X-ray, and electron beam to initiate energy-controlled processes of free-radical polymerization or copolymerization, leading to the production of functionalized, flexible, structured membranes or to the incorporation of functional groups within a matrix composed by a low-cost polymer film. The present manuscript describes the state of the art of using ionizing radiation for the preparation and functionalization of polymer-based membranes for biomedical and environmental applications.

## 1. Introduction

**Figure 1:**
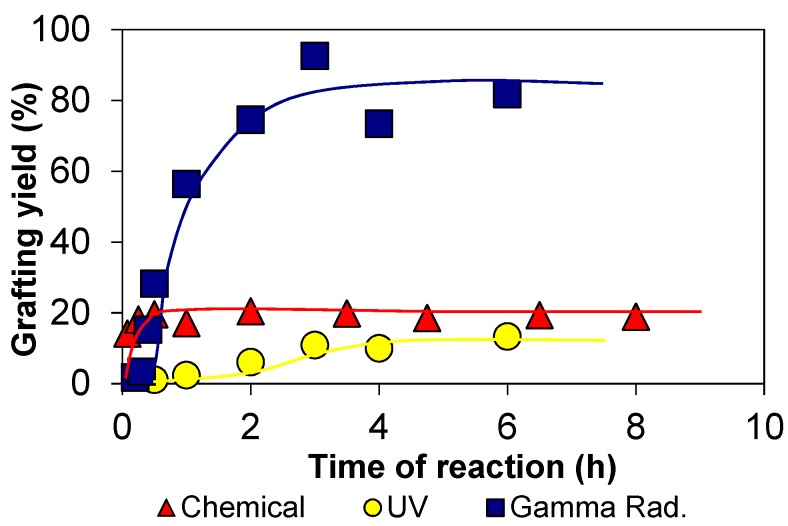
Grafting yields of 2-hydroxyethyl methacrylate (HEMA) graft copolymerization onto chitosan induced by chemical, ultraviolet (UV), and gamma radiation (dose rate 7.4 kGyh^−1^). Reproduced with permission from [3].

Manufactured polymer-based membranes for the most diverse applications in environmental, medicine, and biotechnology fields can be produced by a variety of ways. One important and successful route is by using ionizing radiation processing technologies. Ionizing radiation technologies bring together a unique set of equipment, methods, and procedures through which the molecular structure of materials can be tailored for a particular application on demand and, in this way, can prepare and functionalize new materials. In this type of material processing, no harmful initiators or catalysts are required to initiate the reaction. This ensures that no other compounds than the needed ones are added to the reactional system, lowering the potential for contaminations and impurities [1,2], which can be an issue of crucial importance particularly for electronic and biomedical purposes. In addition, since the absorption of radiation energy generally initiates a free radical process, it is often much more effective than other conventional technologies (Figure 1) [3].

**Figure 2:**
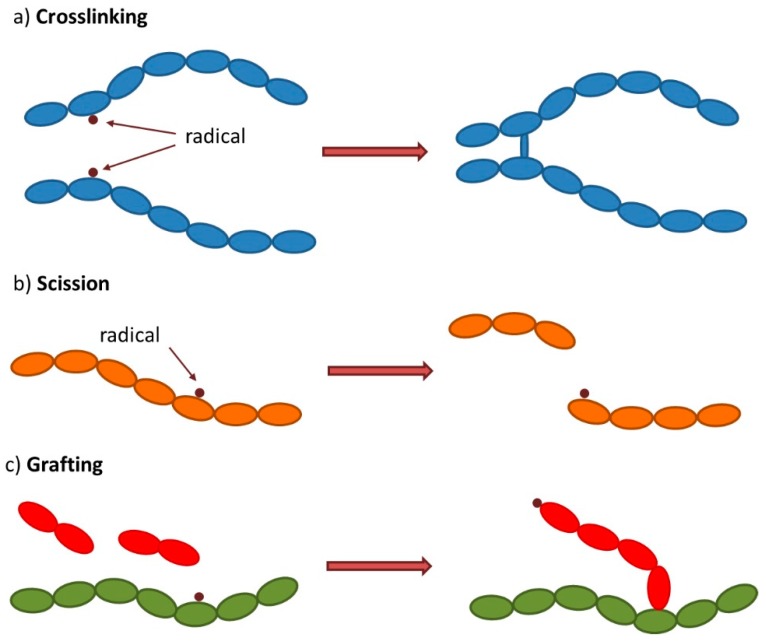
Schematic representation of (**a**) crosslinking, (**b**) scission, and (**c**) grafting reactions induced by ionizing radiation.

Another advantage of radiation processing includes the possibility of polymerizing monomers usually difficult to polymerize by conventional methods. It allows a more reliable reaction control by the appropriate selection of irradiation parameters (absorbed dose, dose rate, and irradiation atmosphere) and the possibility to use different methods such as crosslinking, curing, and controlled degradation by chain scission, degradation, and grafting and to tailor and adequate the final material to the application on demand [4]. The schematic representation of the most relevant methods for membranes preparation/functionalization and obtained structures is depicted at Figure 2.

Ionizing radiation technologies do not require high temperatures, initiators, or solvents and, because of that, are regarded as clean and environmentally friendly. They have been applied over the years in the preparation/modification of polymers, with membranes included, leading to an increased effectiveness and cost reduction of the entire functionalization process. The preparation method can trim the properties of the membranes and can be used to access the desired properties by exploring a variety of methods [5]. The design of innovative membranes by radiation processing technologies thus presents advantages not only in terms of degree of control over the resulting functionalization but also in terms of cost and sustainability.

This manuscript focuses on the use of ionizing radiation for the preparation and functionalization of polymer-based membranes for biomedical and environmental applications. Characterization techniques as well as important concepts will be addressed.

## 2. Ionizing Radiation 

**Figure 3:**
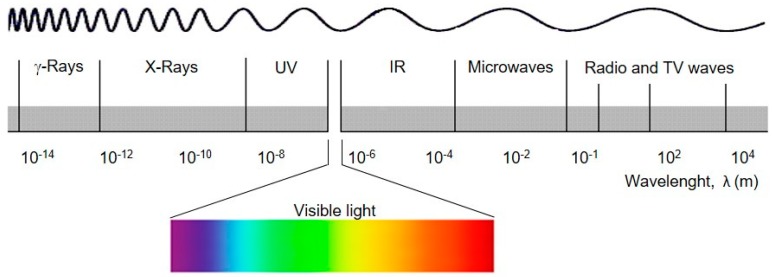
Electromagnetic spectrum.

Considering that the ionization radiation assumes a special role in this manuscript, it is important to try a definition. Ionizing radiation, or high energy radiation, is a term used in general to refer to an electromagnetic wave or to any beam of accelerated particles (electrons or atomic nuclei) which carry enough energy to ionize or remove electrons from an atom or molecule. Considering electromagnetic waves, there are two types that can ionize the medium with which interact: X-rays and gamma-rays. For historical reasons, the term γ radiation is used for the electromagnetic radiation emitted by the nucleus (nuclear transitions between states of different energy) and the term X-rays is used for the radiation emitted in electronic transitions of atoms (transitions between excited and non-excited states with an energy difference on the order of the kiloelectronvolts (keV)). There are no differences in the properties and types of interaction of these radiations with matter. Although in a small range of the electromagnetic spectrum their energies may be similar, in practical applications, these two types of ionizing radiation match different levels of energy. Gamma rays are electromagnetic waves similar to light but in which the energy of the photon is much larger and consequently the wavelength is much smaller (Figure 3) [6].

The penetration power of the radiation depends on its nature and the respective initial energy. Gamma radiation (γ) is a very penetrating electromagnetic radiation arising from the radioactive decay of atomic nuclei like ^60^Co or ^137^Cs. Its energy ranges from a few kiloelectronvolts (keV) to some megaelectronvolts (MeV). Concerning X-rays, this radiation type is produced outside of the nucleus by electrons and their energy ranges from 100 eV to 100 keV. Another constraint factor of radiation penetration range is the type of material with which it interacts in its path. Consequently, even having the same energy, different types of ionizing radiation can penetrate in different ranges of material [6]. Regarding electron beam, its radiation is generally characterized by its small penetration capacity and high dose rates. The irradiation beam, with energies typically between 0.2 and 12 MeV, consists of a high-energy electron flux generated in particle accelerators (electron accelerators). Due to its low penetration power in matter, its application in the field of materials science (property modification) is practically confined to surface treatments and modification of low-density materials [6].

The main effect of the passage of high-energy radiation through a material medium is the ionization of its atoms and molecules, i.e., the release of electrons and the formation of positive ions. However, the transfer of energy to the medium may not have a direct ionizing effect. The energy yielded by radiation to the atomic electron from the substrate may not be sufficient to pull it out of the atom, giving rise only to the appearance of electronically excited species. These processes, independent of the temperature and molecular structure of the material, occur randomly and result in the formation of a first generation of particles, free electrons, positive ions, and excited molecules. From the reaction of these primary products with the medium arises a second generation of active intermediate particles, consisting of excited molecules, positive and negative ions, and free radicals. Together, these new chemical species ensure a very uniform activation of the substrate and the continuity of the subsequent reactions [7].

Considering reactions in aqueous solution, ionizing radiation produces the radiolysis of water, leading to the generation of the •OH, e_aq_^−^, with •H being those species that initiate the reaction chains in the media [8]. In polymers, which is what is mainly concerned when speaking about membranes, ionizing radiation deposits their energy in the media mostly through inelastic collisions with the electrons, leading to processes such as ionization and electronic excitation. It is also possible that ionization leads to the emission of secondary electrons that leads to further ionizations, inducing an electron multiplication effect. Those ionizations drive the appearance of local electric positive charges or holes, which are created after electron ejection [9].

Most of the studies on the interaction of ionizing radiation with polymers, with objectives of preparation of new materials or modification of their properties, have been carried out with electron beams or with γ-radiation.

## 3. Membranes Preparation and/or Functionalization

The underlying idea of using more than one compound in the membrane preparation/functionalization is to take advantage of the properties of both components to optimize structural, mechanical, and functional properties. As an example, the good biocompatibility, nontoxicity, and biodegradability of chitosan, a polysaccharide of natural origin, can be combined with the good mechanical characteristics of the synthetic polymer poly(hydroxyethyl methacrylate), a synthetic polymer. This combination results in the preparation of membranes for biomedical applications [10]. Trying to explore this type of synergy, one of the more relevant methods already mentioned, is the grafting of one material on a backbone of another one by using radiation as an initiator of the process (Figure 2c) [3,11,12,13,14,15,16,17,18]. Due to the availability of a vast number of polymer/monomer combinations and the possibility to combine “incompatible characters” (e.g., hydrophobicity and hydrophilicity), this technique allows the design of advanced functional materials with tailored compositions. The performance of these membranes is thus dependent on the relation between the structural properties and the chemical nature of the components. However, the structural properties are the result not only of the composition and grafting reaction but also of the crosslinking and degradation extension that always occur in radiation-induced reactions. In this way, depending on its extension, particularly in what concerns to crosslinking, this parallel process can also improve the mechanical, chemical, and thermal resistance, giving rise to new properties on the materials such as memory effects, equilibrium swelling, and elasticity, which are of great importance for membranes biological and environmental applications. Beyond that, as there is no different between the type of interaction carried out by electron beam and gamma radiation with matter, the effects on membranes structures will be identical. Nevertheless, as already mention in Section 2, due to electron beam low penetration in matter, electron beam processing is practically confined to surface treatments while gamma radiation interacts at the surface and deeply in matter.

**Figure 4:**
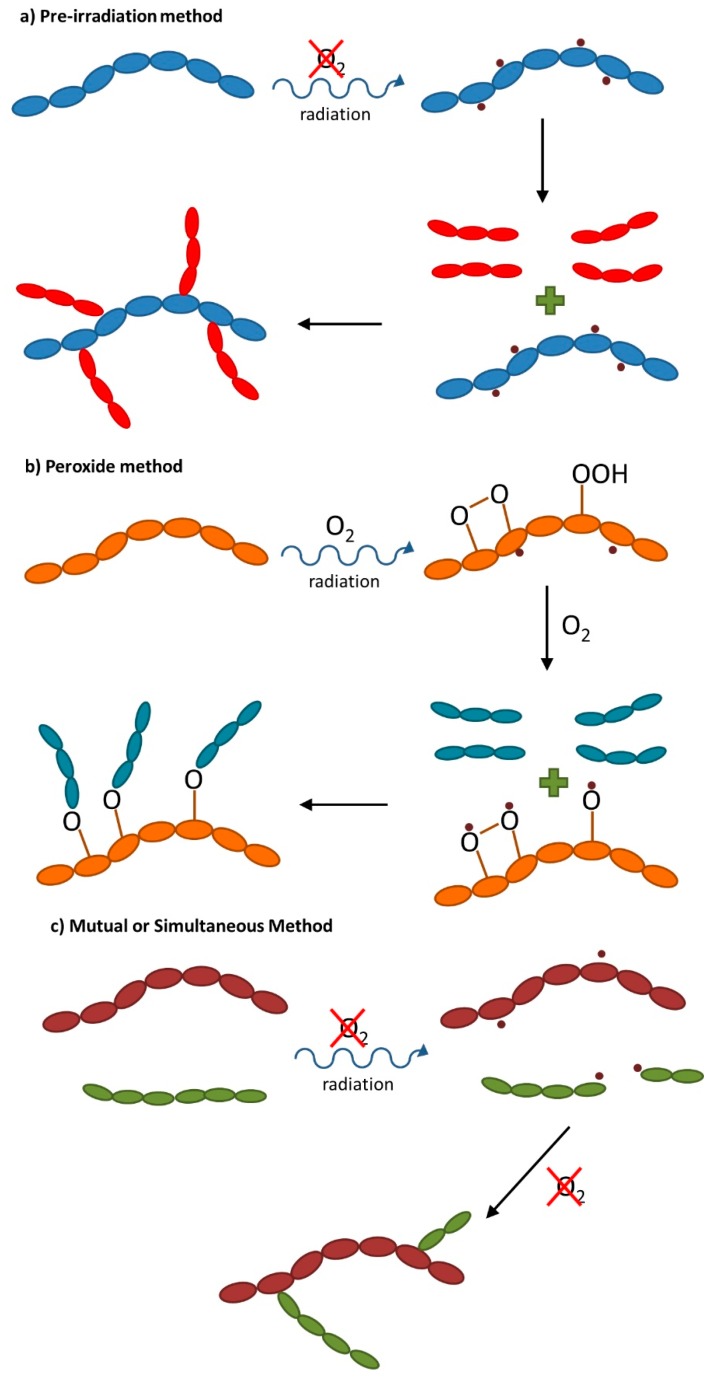
General reactions schemes for radiation-induced graft copolymerization methods: (**a**) pre-irradiation method; (**b**) peroxide method; (**c**) mutual or simultaneous method.

According to Stannet [19], radiation-induced copolymerization can be achieved basically by three distinct methods: (a) the pre-irradiation method, (b) the peroxide method, and (c) the mutual or simultaneous method (Figure 4).

In the pre-irradiation method (Figure 4a), the polymer base is irradiated under vacuum or in an appropriate liquid or gaseous medium prior to contact with a monomer, which may be in liquid or vapor form. The free radicals formed by the radiation get trapped in the polymer and then react with the monomer. As there is no activation of the monomer by radiation, homopolymer formation is minimized. However, as the polymeric substrate is not protected by the monomer, some polymer degradation or crosslinking may occur [20,21]. The efficiency of this complex procedure is dependent on the radical lifetime in polymer substrate. The peroxide method (Figure 4b) is based on the creation of peroxidized species, with the role of intermediate agents in the copolymerization reaction. It is a multistep procedure that usually requires relatively high doses and where the intermediate agent is not more than the peroxidized polymer base obtained by irradiation in the presence of oxygen (air). Peroxidized polymers, which are almost very stable, have the advantage that they can be stored at room temperature until use with a monomer to form the desired copolymer [20,22]. In the case of the mutual or simultaneous method (Figure 4c), the support polymer is irradiated in the presence of the monomer, which may be present as a vapor or liquid or in solution (diluted). Irradiation may take place in air or preferably in an inert atmosphere and results in the immediate formation of active species (mainly free radicals) in the polymer and monomer, which are responsible for the copolymerization (and side homopolymerization) reactions. In addition to its versatility, this method of preparation/functionalization of polymers has good efficiency because the main polymer chain radicals can react as quickly as they are produced. Another advantage is the protective effect that the monomers present in the medium exert on the main polymeric matrix, reducing the undesirable effects of radiation degradation. However, the copolymerization reaction will only be favorable if the number of radicals formed by irradiation on the polymer is greater than the one obtained by irradiation of the monomer (G_value_(polymer) > G_value_(monomer)) [20].

## 4. Membranes Properties and Their Determination

Membrane properties are of the most importance in practical processes and long-term operations. These issues demand a multitask effort regarding the development of adequate methods for membranes preparation that matches the desirable properties, the idealized structural design, practical assembly, and use of the devices. Although the characterization of membrane properties is made according to their nature and intended application, there are some cross-sectional properties as well as several analytical techniques that can be used. In common, all polymers used should go easily through membrane formation with the adequate rigidity and flexibility, chains interactions, steroregularity, and polarity of its functional groups. They can lead to amorphous or semi-crystaline structures, a fact that conditions their performance and applicability. Regarding the industrial fabrication and use of polymeric membranes, the choice of polymers for this purpose have taken into account the reasonable price of polymers used, trying to maintain the low-cost criteria of industrial processes. Most commonly polymers used for membrane preparation are cellulose, nitrocellulose, cellulose acetate, polyamide, polysulfone, poly(ether sulfone), polycarbonate, poly(ether imide), poly(2,6-dimethyl-1,4-phenylene oxide), polyimide, poly(vinylidene fluoride), polyvinyl chloride, polytetrafluoroethylene, polypropylene, polyethylene, polyacrylonitrile, poly(methyl methacrylate), polyvinyl alcohol, and polydimethylsiloxane, and recently, the use of chitosan and other natural polysaccharides has been gaining importance [23].

In the case of membranes for biomedical applications, the emphasis lies mainly in their biocompatibility (hemolytic effect, cytoxicity, etc.), biodegradability, porosity, barrier properties, gas permeability, mechanical properties (flexibility, elasticity, young modulus, etc.), swelling degree, and delivery kinetics in the case of membranes for drug delivery systems. From this set of properties, biocompatibility and biodegradability require specific techniques which are standardized (e.g., ISO 10993) and involves different assays depending on the type of application of the final device. Nevertheless, with a proven efficiency of the material/membrane in in vitro assays, its approval for biomedical applications demands that they have yet to be submitted to in vivo assays, which requires authorization from the national Scientific Ethical Committee on Animal Experimentation. Data regarding membranes biodegradability, namely kinetic of degradation and degradation products, are essential for permanent and nonpermanent membrane applications. It includes the study of mechanical stress and simulated body-chemical environment on degradation processes. Apart from these specific characterization processes involving cells assays and surgical procedures, the characterization of membranes involves the use of common techniques for materials characterization (e.g., Fourier transform infrared (FTIR), thermal analysis, scanning electron microscopy (SEM), nuclear magnetic resonance (NMR), X-ray diffraction (XRD), gas permeability, mechanical analysis, surface contact angle, surface free energy, swelling degree, etc.).

Environmental applications of membranes are mainly associated with energy production and storage as well as with filtration processes and the recovery of pollutants (e.g., ion metals, toxic pollutants, dyes, and treatment of radioactive waste waters). The use of polymeric membranes in catalytic processes for the reuse of waste products (e.g., biodiesel production from used cooking oils) is another growing area of research. For these applications, special attention must be given to membranes chemical selectivity/affinity and transportation vs. permeation properties. Simultaneously, their mechanical resistance, structure (porous distribution and dimensions), chemical stability/reactivity/functionalization (e.g., permeability of penetrant, polymer chain movement, penetrant-polymer interaction, reactivity of functional groups on polymer, etc.) are critical properties for polymeric membranes efficiency on practical long-term operations.

Separation processes are one of the most important and successful applications of polymeric membranes, with high impact in the industry, health and environment. This is particularly relevant concerning the use of ion exchange membranes (IEM). Thus, special attention is given to some of their particular properties and respective determination.

By studying the properties of the membranes, it is possible to infer on ion exchange membranes (IEM) performance under different applications. Thermal and chemical stabilities are indicators of the durability of the membranes. IEMs should be mechanically strong and thermally and chemically stable under operating conditions. In terms of chemical stability, the conditions under which the membranes will be applied and can be as varied as stability to oxidation, reduction, hydrolysis, rehydration, pH, etc. must be present. The thermal stability of IEMs is dependent of the crosslinking degree, intrinsic thermal stability of inert polymers, and reinforcing fabric and the size of the counter-ion. In the case of transport-related properties, such as permselectivity, swelling degree, ionic conductivity, and ion-exchange capacity (IEC) are detrimental to the membrane’s performance and most of them can be determined experimentally, while the rest can be calculated based on other available parameters. It is important to have in mind that the relative importance of these properties vary with the different applications, as will be discussed in the next section.

Water content is of crucial importance to membrane stability and its transport properties. High water content has a positive effect on membrane conductivity; however, it is an indication of a loose mechanical structure that often results in poor permselectivity [24,25]. The water content is influenced by membrane material, fixed charged groups, cross-linking degree of the membrane matrix, and the surrounding solution conditions [26]. The water content of a membrane can be experimentally quantified by measuring the membrane swelling degree (SD) [24]:

Gravimetric determination of the swelling degree (SD) is as follows:

(1) $$\mathrm{SD}=\frac{m_{\mathrm{wet}}-m_{\mathrm{dry}}}{m_{\mathrm{dry}}}$$

where m_wet_ and m_dry_ are the mass of the membrane in wet or dry conditions, respectively.

**Figure 5:**
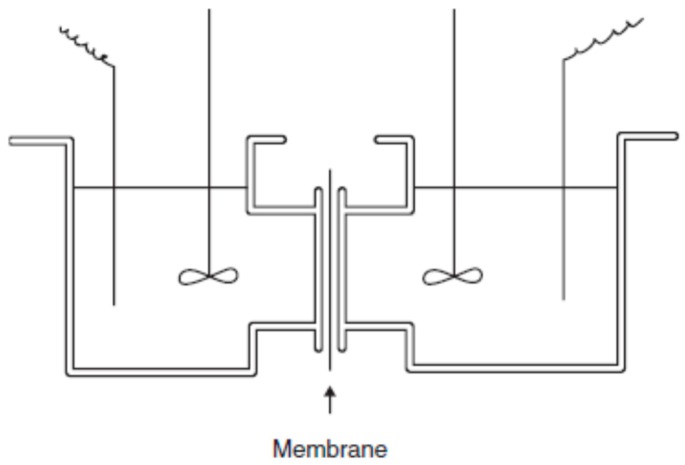
Schematic representation of a cell to determine the permselectivity of a membrane. Reproduced with permission from [27].

Permselectivity measures the flux of a specific component relative to the mass flux through the membrane under a given driving force [24]. In other words, permselectivity of an IEM measures how well the membrane can discriminate between cations and anions. It can be determined by measuring the concentration potential developed between solutions of the same electrolyte at different concentrations separated by the test membrane. Since both potassium and chloride ions have equal mobility, KCl is usually the chosen salt to perform as the electrolyte. An electrolyte with different ion nobilities (e.g., NaCl) would develop a concentration potential even when separated by a nonselective membrane. Additionally, the two electrodes used in the measurement must be identical, which can be verified by measuring the potential between the electrodes when immersed in the same electrolyte (Figure 5). Another way is to take the average value of the potentials during the measurement.

An ideal selective separator develops a potential described by an equation similar to the Donnan equation (Equation (2)) [27]:

(2) $$\Delta V=\frac{RT}{F}\times \ln\frac{C_{1}}{C_{2}}$$

where *F* is the Faraday’s constant, *T* is the absolute temperature, *R* is the gas constant, and C_1_ and C_2_ are the concentrations of the two electrolytes.

Permselectivity, α, is then given by the ratio of the experimental potential (ΔV_exp_) over the theoretical potential (ΔV_theor_) for a membrane with 100% permselectivity (Equation (3)):

(3) $$\alpha =\frac{\Delta V_{\exp}}{\Delta V_{\mathrm{theor}}}\times 100\%$$

Ion exchange capacity (IEC) represents the number of fixed charges, in milli-equivalents (meq) of charged groups, within the IEM per weight of the dry membrane (meq/g membrane) [28]. IEC affects almost all other membrane properties and thus is a crucial parameter of IEMs.

To determine IEC experimentally, it is necessary to turn the cation-exchange membranes (CEMs) into its H^+^ saturated form and the anion-exchange membranes (AEMs) into its Cl^−^ saturated form and then to determine the number of these counter-ions [29]. High values of fixed charges promote membrane swelling, so high values of IEC usually mean high values of swelling degree (SD) [24,30]. Additionally, while on one hand, high IEC tends to increase membrane permselectivity, on the other hand, high SD may adversely affect permselectivity [24,31], and thus, these competing factors lead to a compromise between IEC and SD effects.

The area resistance of a membrane is an important parameter in several electrochemical applications, where selective transport of charged particles is required. For instance, in electrodialysis (ED) processes, the membrane resistance is directly related to the energy consumption and, in the case of reversed electrodialysis (RED), to the maximum power output. Membrane resistance represents the hindrance of an IEM to the ionic current transportation and is a major contribution to the internal resistance, e.g., of a RED stack where higher membrane resistances increases the voltage drop over the RED stack reducing the power output [28]. It is important to have in mind that ion transport through the membrane is performed by ions within the mobile phase, including the counter-ions that compensate the fixed charges at the internal surfaces of membrane pores and the counter-ions pairs of the co-ions in the aqueous phase within the membrane matrix [32]. The mobility of ions tends to be highly sensitive with the water content of the IEM, the concentration of the external salt solution [28,33], as well as the temperature which can increase the ion mobility substantially with higher temperatures [31]. As so, lower membrane resistance is expected for IEMs with higher IEC, salt solution concentration, and working temperatures and, simultaneously, a lower cross-linking degree. As an example, membranes with simultaneously high IEC and high SD usually present a relatively low area resistance and poor permselectivity [34].

In general, membrane resistance is measured via the direct current method (DC), but it can be measured in salt solutions by either DC or alternative current (AC) methods. The most widely used DC method is chronopotentiometry; however, DC measurements do not discriminate between the individual contributions of membrane resistance and the additional resistances of the diffusion boundary layer (DBL) (i.e., the ionic transport resistance in the aqueous layers adjacent to the membrane surface), and the electrical double layer effects [28,35,36]. By the DC method, the voltage drop across the membrane is recorded under a series of current density and the membrane area resistance is determined as the slope of the voltage output vs. current density curve. In order to distinguish between the individual contributions of phenomena occurring at the membrane surface, the AC method electrochemical impedance spectroscopy (EIS) is applied and the pure membrane resistance can be determined. In the EIS method, an alternating sinusoidal current or voltage is applied to the working and counter electrodes, and either the same electrodes or a reference one monitors the response of the system. By performing a fitting to an equivalent circuit, using complex nonlinear least-squares enables to interpret the experimental impedance data [37]. By this method, the geometrical capacitance of the film can usually be neglected but it requires an evaluation of the surface morphology of the IEM and the thickness of the diffusion double layer for the capacitance to be used [38].

The property that correlates the permselectivity with the resistance of a membrane is the fixed charge density (FCD). FCD is defined as the milli-equivalents (MEQ) of charged groups per gram of water within the IEM (meq/g H_2_O) [28] and is determined by the ratio of IEC over SD (Equation (4)) [28].

(4) $$\mathrm{FCD}=\frac{\mathrm{IEC}}{\mathrm{SD}}$$

There is no forthright relationship between membrane resistance and FCD due to the influence of different membrane types and their chemistry [24], e.g., some heterogeneous membranes with lower FCD than homogeneous membranes but which present considerable high resistance because of uncharged interstices and separation charged structures [39]. Increasing the cross-linking degree of an IEM matrix leads to higher FCD resulting in better permselectivity [28].

In order to get higher membrane mechanical strength, the common practice is to increase the crosslinking in the membrane matrix, but it tends to increase membrane resistance [30]. Crosslinking promotes the formation of compact membrane structures and is a promising strategy to control excessive swelling and, in the case of direct methanol fuel cells (DMFCs), the permeability of the IEMs [40]. Cross-linking can improve the tensile strength of the IEMs and is also beneficial for membrane chemical stability. For example, in the case of AAEMs, by increasing the cross-linking of the membranes, the free volume between the main chains diminishes, making more it difficult for the hydroxide ions to access the labile cations and thus increasing the membrane chemical stability [41]. The grafting degree (GD), also called Degree of Grafting (DOG), can be easily determined by gravimetry:

(5) $$\mathrm{GD}=\frac{m_{\mathrm{ag}}-m_{\mathrm{bg}}}{m_{\mathrm{bg}}}$$

where m_bg_ and m_ag_ are the mass of the membrane before and after grafting, respectively.

## 5. Membranes Applications

The increasing knowledge of polymer chemistry, physics, materials science, engineering, and applications allowed the development of efficient membranes preparation techniques which have been providing more sophisticated materials with improved performance for an increasing number of different applications. The interest in this multidisciplinary area of research for environmental and biomedical applications was originated mainly on the needs for drinking water in several regions of the planet and on the confirmed importance of membranes in medical hemodialysis treatments. Presently, most of the industrial applications of membranes for separation and other processes uses polymer-based materials [42], wherein most of membranes are ionomers.

**Figure 6:**
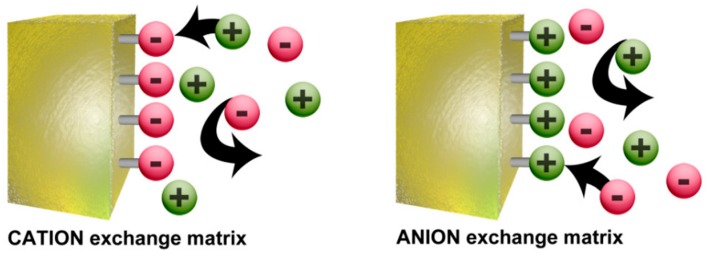
Schematic representation of the Donnan Exclusion effect. Reproduced with permission from [44].

These ionomers are membranes made of co-polymers of which most are nonionic repeating units and a small amount are ionic repeating units (usually less than 15% of the whole polymer). By varying these repeating units of monomers, it is possible to tune the membrane properties to a desired application, making radiation-grafting a suitable method, with high reproducibility for production of large lab-scale batches of different polymer electrolyte membranes, including both cation- and anion-exchange types [43]. In the case of ionomers, it is not surprising that their major applications are directly related with their ion-exchange properties. These ion-exchange membranes (IEM) are classified depending on the charge signal of the ionic repeating units into three categories: cationic, anionic, and amphoteric. When the functional groups of the ionic repeating units are negative, e.g., carboxilates (–CO_2_^−^), sulphonates (–SO_3_^−^), and phosphates (–PO_3_H^−^), etc., then the membrane allows the selective transportation of dissolved cations (defined as counter-ions or mobile-ions) while the anions are retained (defined as fixed ions or co-ions) and the membrane falls in the cationic category. Following the same reasoning, anion-exchange membranes are capable of transporting anions and have positively charged groups, e.g., primary (–NH_3_^+^), secondary (–RNH_2_^+^), tertiary (–R_2_NH^+^), and quaternary (–R_3_N^+^) amino groups, vinylpyridine derivatives, etc. In the case of the amphoteric ion-exchange membranes, both cation- and anion-exchange groups are present in the membrane. The electrostatic exclusion of the co-ions is described by the Donnan Exclusion effect (Figure 6).

The Donnan exclusion effect is based on the incapability of the co-ions to diffuse deeply inside the polymer because the sign of their charge is the same as that of the polymer functional groups. Therefore, ion penetration inside the matrix is governed by the sum of two opposite driving forces: the gradient of the ion concentration and the Donnan effect itself. As an example, in the case of a cation-exchange membrane, the Donnan exclusion effect leads to a flux of anions (mobile-ions) to the membrane and of cations (co-ions) to the solution. This effect results in an accumulation of negative charges in the membrane and positive charges in the solution, giving rise to a difference potential between the two phases called Donnan potential. The Donnan exclusion increases with the increase in Donnan potential, which draws the cations back into the membrane and the anions back into the solution until an equilibrium is established between the electric field and the system’s tendency to eliminate concentration differences by diffusion. Since co-ions are repelled from the membrane, the electrolyte is also repelled because of electro-neutrality requirements. In short, the effectiveness and selectivity of an ion-exchange membrane is related to the overall equilibrium of exclusion-adsorption of ions.

The major parameters that affect an ion-exchange membrane performance, at its various applications, are the concentration of fixed charge in the dry membrane, crosslinking extent, hydrophilic/hydrophobic properties of the polymer matrix, degree and stability of swelling, density of the polymer network, and the distribution of the charge density and the morphology of the membrane itself. No less important is the ion charge density and solution concentration of the electrolyte.

Nowadays, there is an increasing number of commercially available IEMs from several different companies. An important share of the market is accounted by DuPont Co. in Wilmington, DE, USA, ASTOM Co. and Asahi Glass Co., Ltd. in Tokyo, Japan, and FuMA-Tech GmbH in Bietigheim-Bissingen, Germany. The perfluorinated Nafion™ CEMs sold by DuPont Co. are among the most efficient fuel cell membranes and are the benchmark for comparison of new CEMs. Other widely used IEMs for their low resistance, high ion selectivity, and sufficient chemical stability for electrochemical processes are the NEOSEPTA^®^ IEMs produced by ASTOM Co., fumasep^®^ IEMs produced by FuMA-Tech GmbH, and the Selemion^®^ IEMs produced by Asahi Glass Co., Ltd. Additionally, more recently, several Chinese companies, like Shandong Tianwei Membrane Technology Co. Ltd. (Weifang, China), Beijing Tingrun Membrane Technology Co. Ltd. (Beijing, China), and Hefei Chemjoy Polymer Materials Co. Ltd. (Hefei, China), have also started to develop and commercialize IEMs for water treatment and solutions process and are leaders in this field [45]. Other IEMs from other companies are also available, e.g., Aciplex (Asahi Chemicals Co., Tokyo, Japan), Aquivion PFSA (Solavy, Brussels, Belgium), Dais membranes (Dais Analytical Co., Odessa, FL, USA), Flemion (Asahi Glass Co., Tokyo, Japan), Gore-Select (Gore and Associate, Newark, DE, USA), Selemion (AGC Engineering, Mihama-ku, Japan), etc. In a simple search in the Merc Company (former Sigma-Aldrich website, Kenilworth, NJ, USA), one can find just for the perfluorosulfonic acid membrane type at least 42 membranes for fuel cell and electrolysis applications [46].

### 5.1. Biomedical Applications

According to the dictionary, a membrane is “a thin sheet of tissue or layer of cells acting as a boundary, lining, or partition in an organism” (Oxford, 2019). Natural membranes are fundamental for our health and survival, so it is not surprising that, in biomedical applications, artificial membranes can be central either in substituting natural ones [47] or in developing new beneficial uses for our organisms like for instance intra-ocular lenses [48,49].

In terms of biomedical applications, functionalized membranes span a large spectrum of uses like artificial organs (kidney, liver, and pancreas), tissue engineering scaffolds, and drug delivery systems (diffusion-controlled systems, e.g., transdermal ones) [50]. Although the functionalization and synthesis of new materials for those purposes are of great interest, they must pass exhaustive tests to assure that satisfy the requirements for biocompatibility, bio-interaction, and performance. Besides the advantages of ionizing radiation techniques to assure the required chemical, physical, biological, and functional properties, the use of ionizing radiation can even promote an additional advantage by enabling the preparation/functionalization and sterilization in a one single technological step.

#### 5.1.1. Controlled Drug Delivery

**Figure 7:**
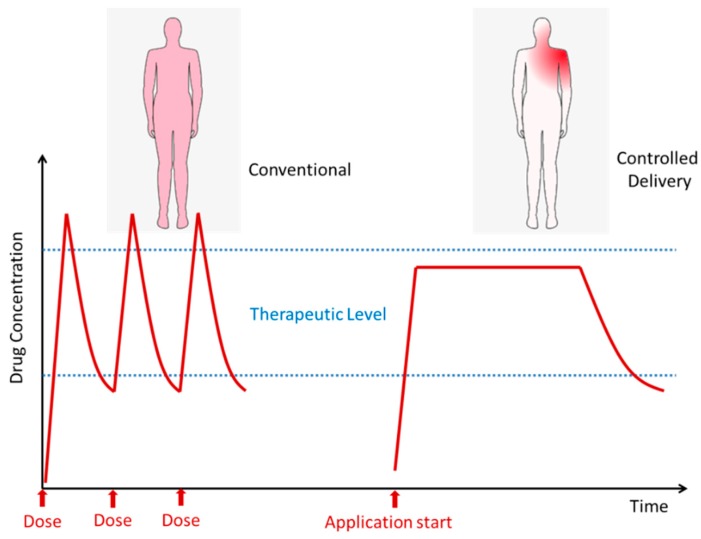
Conventional systemic delivery by doses compared with localized controlled delivery.

One of the important uses of membranes is in the controlled delivery of drugs in several environments. Controlled delivery allows a more stable and localized availability of the desired drugs with a lower global impact of the drug in the organism (Figure 6). The main advantages are fewer doses to be taken by the patient, high period inside the therapeutic level (reducing the risk of toxicity and improving the efficiency), and localized delivery, which prevents other tissues from being affected by the drug. Part of membranes synthesized by ionizing radiation techniques has been reported as been adequate to be used for drug delivery [10,18,51,52,53,54,55,56,57]. However, only some had been tested for real drug delivery studying the permeation of drugs, diffusion coefficients, effect of radiation on the loaded drugs, or rate of drug release [58,59,60,61]. In these works, it can be seen that the release can be extended for long periods in a controlled way, thus avoiding peaks of drugs in the organism. A common factor in the reported cases is the fact that, by controlling the molar ratio, dose rate, and radiation absorbed dose, it was possible to tune the membranes properties and thus develop systems suitable for medical application with regulated drug release (Figure 7).

#### 5.1.2. Tissue Regeneration

The use of membranes for tissue regeneration is not a new subject, having been addressed in several researches at least for the past two decades for the reconstruction and regeneration of wounded tissues as skin, nerves, cartilage, and bones [62,63,64,65]. However, membranes produced to be used for tissue regeneration using irradiation on their preparation are far less common. A number of technical approaches have been applied for scaffold production. In these cases, scaffolds should not only be a substitute of the extracellular matrix but also act as a membrane able to act as a delivery vehicle for cells and/or to serve as a carrier for growth factors [5,66,67,68,69,70,71,72]. The final focus is the same: to engineer membranes with adequate properties able to improve interactions with the cells and to enhance tissue regeneration. However, by using radiation, either as the initiator or as an agent to trim the properties of the final membranes, one can have the additional advantages of avoiding the addition of reagents with potential problems for the final application together with the possibility of sterilization in just one step.

### 5.2. Environmental Applications

Global energy demand is supported mainly by the production of electricity from fossil fuels and nuclear power plants. Since the start of the industrial revolution that started the exploitation of fossil fuels on a large scale, the concentration of CO_2_ in the atmosphere has raised from 280 ppm to more than 411 ppm by May 2019 [73]. The consequent climate changes have led to a change in energy policies, nowadays focused on more “green” and renewable sources, such as wind power, hydroelectricity, and solar and geothermal energy. Because of temporal deviations in energy production and consumption, only a smart grid combining renewable power sources and energy storage systems can answer the turnaround in energy policies [74,75]. Thus, the importance and pace of research on electrochemical energy storage and production increased dramatically [76] and two of the major applications of radiation-grafted membranes are in energy storage and energy production. The other major application of these membranes is in water treatment.

#### 5.2.1. Energy Production

##### Fuel Cells

William Grove discovered the basic operating principle of fuel cells in 1839. The principle that remains unchanged today consists of reversing water electrolysis to generate electricity from hydrogen and oxygen. Fuel cells are electrochemical devices that converts chemical energy into electric energy (and some heat) for as long as fuel and oxidant are supplied. Unlike engines or batteries, fuel cells do not need recharging, and when the fuel used is hydrogen, they generate only water and power. Thus, they can be a so-called zero-emission engine [77]. Additionally, fuel cells have very high current densities and high energy per volume and per weight when compared to other conventional power sources. Furthermore, they present high efficiency conversion because of fewer limitations imposed by the second law of thermodynamics. As so, fuel cells are very promising energy conversion devices to power stationary, mobile, and portable applications in the 21st century [43].

**Figure 8:**
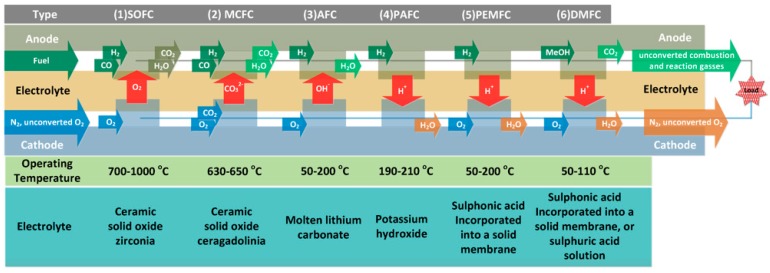
Schematic representation of the fuel cells categories and respective operating temperatures and electrolytes used. Reproduced with permission from [78].

Fuel cells can be classified into several categories: (1) solid oxide fuel cells (SOFC), (2) molten carbonate fuel cells (MCFC), (3) alkaline fuel cells (AFC), (4) phosphoric acid fuel cells (PAFC), (5) proton exchange membrane fuel cells (PEMFC), and (6) direct methanol fuel cells (DMFC) [78]. A schematic representation of the 6 types of fuel cells and the respective temperatures of operation and electrolytes is presented in Figure 8. They are all based on similar working frameworks, with the consumption of fuel (hydrogen or methanol) and oxidant, producing water or CO_2_.

Although many achievements have been accomplished in the development of PEMs and AEMs for application in fuel cells, there is still a need for more improvement, which has prompted researchers to develop new IEMs, giving rise to an increasing number of publications and several reviews [41,43,78,79,80,81,82,83].

##### Proton-Exchange Membranes Fuel Cells (PEMFC)

**Figure 9:**
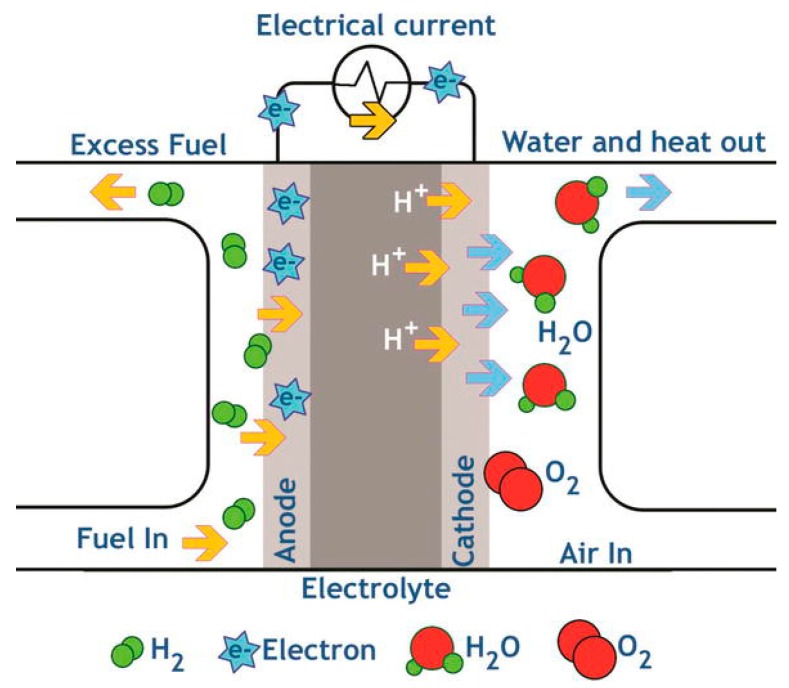
Schematic representation of a proton-exchange membrane fuel cell (PEMFC). Reproduced with permission from [85].

Proton-exchange membranes (PEMs) are semipermeable cation-exchange membranes that simultaneously conduct protons and function as electronic insulators and reactant barriers. Thus, PEMs are a key part of membrane electrode assemblies, like proton-exchange membrane fuel cells (PEMFC), or redox flow batteries (RFB) where they are responsible for the separation of the reactants and transport of protons while blocking a direct electronic pathway through the membrane. PEMFCs are in the vanguard of fuel cell technologies under development [84], and a schematic representation of its operation is represented in Figure 9.

In 1957, Chen et al. reported for the first time cation-exchange studies on a membrane prepared by the radiation grafting technique [86]. This membrane was prepared by the radiation grafting of styrene (St) on a polyethylene (PE) film, and since then, several radiation-grafting processes and functional monomers have been studied. The most studied cation-exchange groups are carboxylic acid and sulfonic acid groups. Of these two, the sulfonic acid groups are more acidic and are currently the ideal proton-conducting groups because of the value of p*K*a ≤ 1, which is lower than the p*K*a = 2–3 values of the carboxylic acid groups. Among the commercial proton-exchange membranes, the perfluorinated Nafion membranes (from DuPont) stand out. They have been commercially available for about 50 years and have been thoroughly studied. The Nafion-series membranes are obtained by the copolymerization of variable amounts of unsaturated perfluoroalkyl sulfonyl fluoride with tetrafluoroethylene, they present high conductivities, and their Teflon-like backbone structure confers them with a long-term stability under both oxidative and reductive environments [43]. Despite the fact that Nafion membranes have been the benchmark to compare newly developed membranes, they present some limitations like poor performances at low humidity (below 80% relative humidity (RH)) or elevated temperatures (above 80 °C) and crossover of vanadium ions and methanol (when applied to redox flow batteries (RFB) and in direct methanol fuel cells (DMFCs), respectively) and are considered expensive.

Recently, Prakash and coworkers made nanochannels in a polymer thin film, employing irradiation of high-energy swift heavy ions (SHIs), followed by selective etching and chemical grafting [87]. The membrane presented a proton conductivity of 4.59 × 10^−2^ Scm^−1^ at 30 °C, while methanol permeability was inferior to that of standard Nafion 117, an indication that it would present a better performance when applied as a fuel cell membrane (1.02 × 10^5^ Scm^−3^s compared to the value of 0.73 × 10^5^ Scm^−3^s for Nafion 117). The membrane electrode assemblies (MEA) studies presented for the functionalized nanohybrid a value of 0.76 V open circuit voltage, a power density of 92 mWcm^−2^, and current density of 252 mAcm^−2^, higher than for Nafion 117.

Sadegui S. et al. reported the preparation of the highly conductive PEM poly(vinylidene fluoride)-graft-poly(styrene sulfonic acid) (PVDF-g-PSSA) membranes [88]. The membranes were obtained by a single-step radiation grafting of sodium styrene sulfonate (SSS) to PVDF in the powder form followed by solvent evaporation methods. The solvent evaporation, at the end of the synthesis, led to the arrangement of submicron-structured ionic channels within the membrane, leading to an increase of proton conductivity with the increase of the grafting level. At 35% graft level, the membranes presented proton conductivities ≈70 mS cm^2^, leading to a power density of 250 mWcm^−2^ at 650 mAcm^−2^.

Nowadays, there is still a need for a solid understanding of how structural and electrochemical processes are affected during PEMFC operation. To this end, Meyer Q. and coworkers recently proposed a comprehensive roadmap for in situ and operando characterization of PEMFCs [89]. This roadmap is an answer to the present lack of knowledge of structure-to-electrochemical performance relationships during operation and tries to answer how these processes correlate to uneven degradation of different areas of the cell.

##### Anion-Exchange Membranes Fuel Cells (AEMFC)

In the last decade, there has been an increasing research of anion-exchange membrane fuel cells and, consequently, an increase in the research of alkaline anion-exchange membranes (AEM). The interest in development of AEMFCs is due to the significant potential advantages they present over traditional proton-exchange membrane fuel cells. Among these, probably the most important one is the possibility of employing low-cost non-Pt-group electrocatalysts for the oxygen reduction reaction (ORR) [90,91]. Additionally, the high pH working conditions allows the use of membrane and cell components (e.g., thin, easily stamped metal bipolar plates) with lower costs due to the less corrosive working environment compared with the superacid media in PEMFCs [92,93].

**Figure 10:**
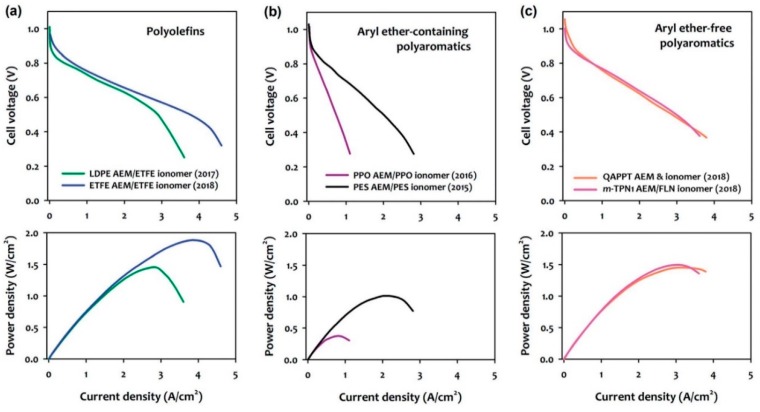
Comparison of H_2_/O_2_ AMFC performances employing (**a**) polyolefins, (**b**) aryl ether-containing polyaromatics, and (**c**) aryl ether-free polyaromatics. Reproduced with permission from [99].

However, AEMFCs still present limited performances and handicaps even when using precious metal catalysts. The search for appropriate ionomers with good stabilities for assembling compact membrane electrode assemblies (MEAs) is still needed. These ionomers are required to exhibit excellent solubility in low-boiling water-soluble solvents, to be alkaline stable, and to present high hydroxide conductive ionomers [45]. In the early 2010s, the performance of the AEMFCs was low, with peak power densities <200 mWcm^−2^ [94]. At the time, Wenchao Sheng et al. [95] suggested that slow hydrogen oxidation reaction (HOR) kinetics was a possible reason for this low performance. Consequently, the research focused on the development of highly active HOR catalysts and the understanding of the HOR mechanistic aspects [96] and were often used to discuss the performance of AEMFCs. Another reason that limits AEMFCs performance is the adsorption of phenyl groups from the membrane ionomers onto the HOR catalyst [95]. The adsorption of benzene and other aromatic compounds on Pt surfaces is well known [91,97] and may play a significant role since all currently available polymer electrolytes contain phenyl groups. In the case of the previously discussed PEMFCs, the impact of phenyl adsorption on Pt has not been an issue because perfluorinated polymer electrolytes without phenyl groups are predominant [98]. These phenyl group adsorptions can be even more significant as state-of-the-art ammonium-functionalized aryl ether-free polyaromatic ionomers recently reviewed by E.J. Park and Y. S. Kim [99] show excellent chemical stability under high pH conditions. Although the best performances of MEAs employing aryl ether-free polyaromatic electrolytes is found to be up to c.a. 1.5 Wcm^−2^ under H_2_/O_2_ conditions at 80 °C, better than the MEAs with aryl ether-containing polyaromatics, it is less than the maximum obtained with a state-of-the-art radiation-grafted polyolefin that reach c.a. 2.0 Wcm^−2^ [99] (Figure 10).

AEMs prepared by radiation-grafted methods have been developed by employing several strategies including the radiation-grafting of vinyl monomers onto non-fluorinated (e.g., low-density polyethylene (LDPE)) [100,101], partially fluorinated (e.g., ETFE [102] and PVDF [103]), and fluorinated (fluorinated ethylene propylene (FEP)) [104] films followed by amination to yield anion conducting materials. Vinylbenzyl chloride (VBC) is a monomer ideal for the preparation of AEMs because of the combination of the reactive –CH=CH_2_ and –CH_2_Cl functional groups. However, VBC is expensive, potentially mutagenic, and acutely toxic when used in large quantities. As so, it is required to reduce to a minimum the quantity of VBC monomers used in the grafting step. The most common method is the dilution of VBC monomers with organic solvents like propanol [105,106], toluene [107], or even propan-2-ol which allowed S.D. Poynton and J.R. Varcoe to lower the VBC concentration down to 20 vol% for grafting onto an ETFE film irradiated with an electron-beam up to 70 kGy of absorbed dose [100]. Following these results, the same research group together with coworkers were able to further enhance these results with the substitution of the diluent organic solvent by water [96]. In this way, they were able to reduce both the amount of VBC monomers and the electron-beam-absorbed dose to 5 vol% VBC and 30–40 kGy, respectively. The decrease in the radiation doses resulted in mechanically stronger AEMs. Additionally, the use of water leads to a more uniform distribution of the VBC grafts, leading to an increase in Cl^−^ conductivities (up to 68 mScm^−1^ at 80 °C for the fully hydrated AEMs in comparison to the 48 mScm^−1^ for the AEM prepared with propan-2-ol).

**Figure 11:**
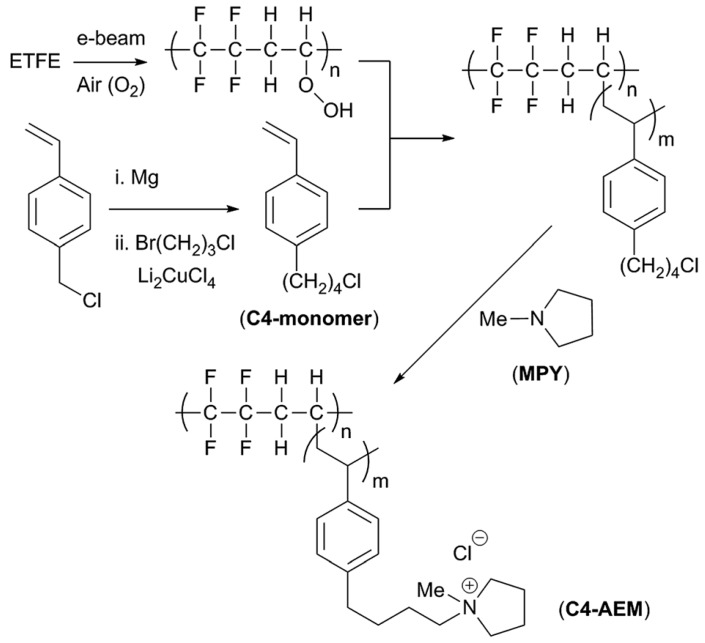
Schematic outline of the synthesis of the pyrrolidinium-grafted-ETFE-based C4-AEM: The methylene benchmark contained a single –CH_2_– group between the benzene ring and the pyrrolidinium. Reproduced with permission from [112].

As already stated, one of the major problems of AEMs is alkaline stability. Due to the easiness of polymerization and functionalization of aromatic ionomers, they have been widespread used in AEMs. However, cationic head-groups covalently bound to benzylic positions are sensitive to hydroxide substitution [108,109,110]. A strategy to reduce the degradation is the introduction of an aliphatic spacer between the aryl and the cationic head-groups. This strategy, reviewed a few years ago [111], not only improves the membrane stability but also usually increases OH^‒^ conductivity. An example was recently published by J. Ponce-González and co-workers whom, by the addition of a butyl-spacer between the benzene and the methylpyrrolidinium groups in an ETFE-based radiation-grafted AEM, doubled the ex situ alkali stability at 80 °C (when compared to the methylene benchmark) (Figure 11) [112]. Although, in this case, the obtained lower degree of grafting (in comparison with the methylene benchmark) led to a decrease in conductivity, they could still get peak power densities above 1 Wcm^−2^.

J. Ponce-González and coworkers performed a systematic study on different cationic head-group chemistries on several ETFE-based radiation-grafted AEMs [113]. The relative comparison of a series of benzyl-linked saturated-heterocyclic quaternary ammonium (QA) radiation-grafted ETFE-based AEMs showed that the benzyl-N-methylpyrrolidinium one presented the highest alkali stability, conductivity, and in situ fuel cell performance even in comparison to the prior benzyltrimethylammonium benchmark. Later, a series of three AEMFCs were assembled with AEMs prepared with irradiated ETFE-based AEI (anion-exchange ionomer) powders with the QAs benzyl-*N*-methylpiperidinium (MPRD), benzyltrimethylammonium (TMA), and benzyl-*N*-methylpyrrolidinium (MPY) showing very good performances at 60 °C [107]. However, at the same time, a study with a new high-density polyethylene-based (HDPE) radiation-grafted AEM proved that using HDPE as a precursor film directly led to enhanced performance characteristics in comparison to an ETFE one [114]. Nevertheless, the longest durability testing of an AEMFC (H_2_/O_2_-fed) was reported by Omasta et al. [115] using radiation grafted ETFE films bearing benzyltrimethylammonium (BTMA) cations as the AEM. In this work, the authors created new electrode compositions by the systematic change of ionomer and carbon content in the anode catalyst layer and the AEMFCs fabricated with a balanced AEI:C:Pt ratio at the anode were able to operate for more than 400 h. During this time, the cell voltage declined 40% during the initial 100 h of testing with only minor voltage decay over the last 300 h and a performance recovery after a simulated 8 h cold shutdown. These gains were achieved by improving the water mass transport behavior of the AEMFC. These balanced anode designs led to a water management so efficient that mass transport is no longer the dominating loss within the cell but it is the ohmic resistance. This improvement in the mass transport behavior allowed for the observation of the records for achievable mass transport limiting current of 5 Acm^−2^, current density at peak power of 4 Acm^-2^, and a record peak power density of 1.9 Wcm^−2^.

#### 5.2.2. Energy Storage Devices

Conventional power plants, based on fossil fuels, as well as nuclear power plants produce a stable and on-demand load of electricity. However, as stated above, climate changes have led to a change in energy policies, an increasing number of new power plants using solar energy and wind power were built around the world, and a major expansion is planned until 2020 (solar) and 2050 (wind) [116]. Additionally, small-scale photovoltaic energy production is also increasing [117]. Never as today was the production of electricity so dependent on the weather and thus generated in a discontinuous way and in an increasing decentralized manner. As so, demand can often diverge from energy production, increasing the need for large-scale electrical energy storage devices for grid-scale storage within smart grids to overcome temporal deviations in energy production and consumption. In this regard, Redox Flow Batteries (RFBs) have been intensively studied for diverse capacity and power needs of up to MW/MWh.

**Figure 12:**
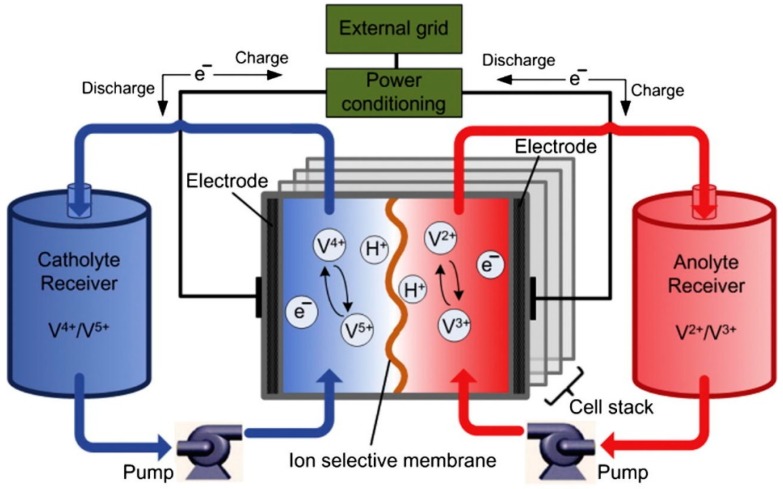
Schematic outline of a vanadium redox flow battery (VRFB). Reproduced with permission from [118].

The main components of RFBs are an electrochemical cell and two tanks. The cell contains two electrodes separated by a membrane (Figure 12). The redox-active cathode and anode materials are dissolved in the electrolyte and, because of that, are named as either the catholyte or anolyte.

As can be deduced from Figure 11, polymer electrolyte membranes (PEMs) are a key component of rechargeable RFBs. These membranes are responsible for both the separation of the catholyte from the anolyte and of proton conductivity. As so, these membranes need to present high proton conductivity; low electron conductivity; low crossover of the two electrolytes; and good thermal, mechanical, and chemical stability. Additionally, easy handling and low costs are required for technical applications. Some recent publications present the preparation of cationic, anionic, and amphoteric specifically radiation-grafted [76,80,119] or more generalized [120] membranes for RFBs.

Among RFBs, vanadium redox flow batteries (VRFB), also commonly known as all-vanadium redox flow batteries, are the most studied ones [76,121] because of their excellent features like relative low cost, long life, easy operation, and deep discharge capability, as well as high current efficiency. Not surprisingly, in sight of its similarities with fuel cells, commercial Nafion membranes are used in VRFBs because of its good proton conductivity and high chemical and mechanical stability [121] and are the benchmark for the development of alternative membranes [116,119]. These membranes present however the limitations of VO_2_^+^ ion crossover and are expensive (around 40% of the total cost of the cell). Vanadium crossover lowers the efficiencies, leading to an increase of the self-discharge rate of the battery. Recently, Jiang, B. and coworkers reported a study the Nafion series membranes application in RFBs [122]. Other types of CEMs containing sulfonic acid groups were developed due to their excellent proton conductivity and their mechanical and chemical stabilities. However, a high degree of sulfonation is required to obtain high conductivity, which often leads to high swelling ratio and reduced mechanical stability [123].

This year (2019), Cui, Y. et al. prepared a series of amphoteric membranes by preirradiation-induced graft copolymerization of styrene and dimethylaminoethyl methacrylate in an aqueous emulsion media followed by solution casting, sulfonation, and protonation. Among the poly(vinylidene difluoride)-based membranes prepared, the membrane with a grafting yield (GY) of 28.4% presented higher IEC and conductivity as well as lower permeability of VO_2_^+^ ions and maintained longer times to open circuit voltage than the benchmark Nafion 115 [124].

Abdiani, M. and coworkers, recently reported a series of new polyolefin-based alkaline PEMs. These membranes were prepared in a three-step procedure of radiation-graft copolymerization of poly(vinylbenzyl chloride) (PVBC) onto polyethylene/polypropylene (PE/PP) followed by conversion into dense membranes and finally the introduction of quaternary amine groups. The prepared series of amphoteric membranes showed high anion conductivity, good chemical, and mechanical stabilities with low vanadium crossover (permeability). In comparison with Nafion 117 benchmark, the PE/PP-g-PVBC-TMA membranes presented superior performance due to the 16-times reduction of vanadium ion permeability and energy efficiency enhanced by 9% at current density of 80 mA cm^−2^. This performance was explained by the presence of positively charged ionic groups in the membrane structure.

#### 5.2.3. Water Treatment

In the use of polymeric-based membranes functionalized by ionizing radiation for water treatment applications, studies reported in literature show that they are mostly designed for separation and purification purposes [125]. For instance, it is possible to find several examples referring to the removal of heavy metal ions from industrial wastewater as well as other toxic pollutants from water/wastewater [126,127,128,129,130,131,132,133]. For example, Bazante-Yamaguishi et al. [134] have compared the use of electron beam and gamma radiation to functionalize polypropylene (PP) and polyvinyl chloride (PVC) substrates to produce an aluminum-selective material to improve the quality of water resources. Although scarce, some other applications include ultra-pure water production as the ones mentioned by Saito and coworkers [135] that tested radiation-grafted sulfonic acid porous hollow-fiber membranes with strong affinity for Na^+^ ions and high water permeability. Takeda et al. [136] used thermally adhered high-density polyethylene grafted with glycidyl methacrylate to obtain an ion exchange fabric also for ultra-pure water production. Other applications like desalination of seawater have also been reported [137,138] but present a smaller contribution to industrial applications mainly due to economic reasons. Additionally, there are some researches in literature that refer to processes based in ultrasonic radiation or sonication [139,140].

## 6. Conclusions

Radiation processing has long been recognized as an energy-efficient technology with strong and promising perspectives in several areas of application. The specific advantages of this technology are being used for the development of new materials for applications in such different areas as medicine and healthcare, electronics, energy, environment, advanced materials, food, and agriculture.

These last advances in polymer science and technology resulted mainly from the development of atomic and spatial technology in the forties of the last century which, beyond many other technological developments, led to the creation of processes for the safely and effective modification of the properties of polymeric materials by radiation. In the last part of the sixties of the twentieth century, the spread of radiation technologies to industry had already been a successful reality mainly concerning the materials processing and development.

As it was illustrated in this review, radiation processing of materials can be controlled and successful used for the development of novel materials with tailored properties for specific and sensitive applications. These applications, with high industrial interest, simultaneously constitute an asset in terms of human well-being and more environmentally friendly practices.

The main advantages of radiation processing of advanced polymeric materials can be resumed as (i) the availability of different radiation sources and the control of the irradiation parameters (e.g., dose rate, irradiation environment, and method of irradiation) to enable the adjustment of reaction parameters such as the initiation and propagation velocity as well as the depth extension of the desired modification; (ii) the processes are normally carried out at room temperature and without the use of catalysts, being almost completely free of polluting and/or highly toxic solvents in most cases; (iii) high reaction yields associated with simpler and more efficient isolation operations; and (iv) final products with high purity and homogeneity of properties obtained through a less polluting way and with considerable energy and resources savings.

This set of characteristics, associated with the sustainability already demonstrated by the radiation technologies, allow its framing despite the restrictions from “radiation skeptics” in the group of green chemistry techniques. The modification and control of properties of polymeric materials by ionizing radiation processing is a multidisciplinary area of research which encompasses knowledge from polymer science, chemistry, radiation chemistry, radiation physics, medicine, biology, tissue engineering, and materials science. Complementary new “bio-inspired” areas of research appear mainly due to the development of new polymeric materials for biomedical applications: bioelectronic (biomolecular electronic) and bioinformatic.

Concerning some of environmental applications, the most relevant properties studied in the membranes are high ion-exchange capacities, high proton conductivities, low methanol transport, reasonable water uptake, and low gas permeability. However, in the case of radiation-grafted membranes for fuel cells, there are still several parameters that remain to be solved before many of these membranes can pass from the laboratory to practical applications. The main challenge is the stability and durability improvement under dynamic conditions, since fuel cells require more than 5000 h of continuous operation.

To sum up, from the work presented within, it is possible to realize that radiation technologies have an enormous potential as a tool for the development and tailoring of new polymer-based materials and that “this story is almost in the beginning”.

## Abbreviations

AEI	anion-exchange ionomer
AEM	anion-exchange membrane
AEMFC	anion-exchange membranes fuel cells
AFC	alkaline fuel cells
AIMFC	amphoteric ion-exchange membranes fuel cells
BTMA	benzyltrimethylammonium
CEM	cation-exchange membranes
DBL	diffusion boundary layer
DOG	degree of grafting
DMAEMA	dimethylaminoethyl methacrylate
DMFC	direct methanol fuel cells
EIS	electrochemical impedance spectroscopy
ETFE	poly(ethylene-co-tetrafluoroethylene)
FCD	fixed charge density
FEP	fluorinated ethylene propylene
GD	grafting degree
GY	grafting yield
HEMA	2-hydroxyethyl methacrylate
HDPE	high-density polyethylene
HOR	hydrogen oxidation reaction
IEC	ion-exchange capacity
IEM	ion-exchange membrane
LDPE	low-density polyethylene
MEA	membrane electrode assembly
MCFC	molten carbonate fuel cells
MPRD	benzyl-N-methylpiperidinium
MPY	benzyl-N-methylpyrrolidinium
ORR	oxygen reduction reaction
PAFC	phosphoric acid fuel cells
PE	polyethylene
PEM	polymer electrolyte membrane
PEMFC	proton-exchange membranes fuel cells
PFSA	perfluorinated sulfonic acid
PHEMA	poly(hydroxyethyl methacrylate)
PP	polypropylene
PVBC	poly(vinylbenzyl chloride)
PVDF	polyvinylidene difluoride
PSSA	poly(styrene sulfonic acid)
QA	quaternary ammonium
RFB	redox flow batteries
RG	radiation-grafted
SD	swelling degree
SGE	salinity gradient energy
SGP	salinity gradient power
SHIs	swift heavy ions
SOFC	solid oxide fuel cells
SSS	sodium styrene sulfonate
St	styrene
TMA	benzyltrimethylammonium
VBC	vinylbenzyl chloride
VRFB	vanadium redox flow battery
